# Effects of Spatial Variability and Relic DNA Removal on the Detection of Temporal Dynamics in Soil Microbial Communities

**DOI:** 10.1128/mBio.02776-19

**Published:** 2020-01-21

**Authors:** Paul Carini, Manuel Delgado-Baquerizo, Eve-Lyn S. Hinckley, Hannah Holland‐Moritz, Tess E. Brewer, Garrett Rue, Caihong Vanderburgh, Diane McKnight, Noah Fierer

**Affiliations:** a Cooperative Institute for Research in Environmental Sciences, University of Colorado, Boulder, Colorado, USA; b Departamento de Biología y Geología, Física y Química Inorgánica, Escuela Superior de Ciencias Experimentales, Universidad Rey Juan Carlos, Móstoles, Spain; c Institute of Arctic and Alpine Research, University of Colorado, Boulder, Colorado, USA; d Environmental Studies Program, University of Colorado, Boulder, Colorado, USA; e Department of Ecology and Evolutionary Biology, University of Colorado, Boulder, Colorado, USA; Pacific Northwest National Laboratory

**Keywords:** microbial seasonality, soil microbial ecology, microbial interactions, soil bacterial and fungal communities, hillslope aspect, nitrogen cycling, Critical Zone Observatory Network

## Abstract

Few studies have comprehensively investigated the temporal variability in soil microbial communities despite widespread recognition that the belowground environment is dynamic. In part, this stems from the challenges associated with the high degree of spatial heterogeneity in soil microbial communities and because the presence of relic DNA (DNA from dead cells or secreted extracellular DNA) may dampen temporal signals. Here, we disentangle the relationships among spatial, temporal, and relic DNA effects on prokaryotic and fungal communities in soils collected from contrasting hillslopes in Colorado, USA. We intensively sampled plots on each hillslope over 6 months to discriminate between temporal variability, intraplot spatial heterogeneity, and relic DNA effects on the soil prokaryotic and fungal communities. We show that the intraplot spatial variability in microbial community composition was strong and independent of relic DNA effects and that these spatial patterns persisted throughout the study. When controlling for intraplot spatial variability, we identified significant temporal variability in both plots over the 6-month study. These microbial communities were more dissimilar over time after relic DNA was removed, suggesting that relic DNA hinders the detection of important temporal dynamics in belowground microbial communities. We identified microbial taxa that exhibited shared temporal responses and show that these responses were often predictable from temporal changes in soil conditions. Our findings highlight approaches that can be used to better characterize temporal shifts in soil microbial communities, information that is critical for predicting the environmental preferences of individual soil microbial taxa and identifying linkages between soil microbial community composition and belowground processes.

## Abstract (precis)

Nearly all microbial communities are dynamic in time. Understanding how temporal dynamics in microbial community structure affect soil biogeochemistry and fertility are key to being able to predict the responses of the soil microbiome to environmental perturbations. Here, we explain the effects of soil spatial structure and relic DNA on the determination of microbial community fluctuations over time. We found that intensive spatial sampling was required to identify temporal effects in microbial communities because of the high degree of spatial heterogeneity in soil and that DNA from nonliving sources masks important temporal patterns. We identified groups of microbes with shared temporal responses and show that these patterns were predictable from changes in soil characteristics. These results provide insight into the environmental preferences and temporal relationships between individual microbial taxa and highlight the importance of considering relic DNA when trying to detect temporal dynamics in belowground communities.

## INTRODUCTION

Information on the temporal dynamics of microbial communities over different time scales can be used to better understand the factors influencing the structure of microbial communities and their contributions to ecosystem processes. The microbial communities found in the human gut (1), leaf litter (2), marine (3), and freshwater (4) habitats can exhibit a high degree of temporal variation. Although the magnitude and timing of this temporal variation in community composition can vary depending on the environment and taxon in question, such temporal variability is often predictable from environmental factors (5). For example, ocean microbial communities display predictable periodic oscillations over time (seasonality) that have been linked to regular changes in biotic and abiotic factors, including phytoplankton dynamics and physicochemical factors (reviewed in references 3 and 6). These changes in environmental conditions influence the nature of biotic interactions within these ecosystems and can have important ramifications for understanding the functional attributes of microbial communities and the ecosystem services they provide (7–9).

Understanding how temporal changes in environmental conditions influence soil microbial communities is necessary to accurately understand how microbial communities contribute to soil processes and for using microbes as bioindicators of changes in belowground conditions such as carbon and nutrient availability—parameters that are often difficult to measure directly. However, to understand temporal variation in soil microbial communities, we need to consider spatial effects due to the highly structured nature of soil and the destructive nature of soil sampling. Thus, contextualizing how temporal dynamics in microbial community structure relate to small-scale spatial variation may help us better understand the factors controlling microbial community structure and the processes microbes mediate. Results from previous studies of temporal variability in soil microbial communities are idiosyncratic. While some studies show that soil microbial communities exhibit measurable temporal variation in response to experimental warming (10, 11) and seasonal patterns in temperature and moisture (12–16), other studies show no or minimal variation in microbial communities over time, despite marked changes in environmental conditions (5, 17, 18). One possible explanation for the discrepancies across studies is that the spatial heterogeneity in soil microbial communities can be sufficiently large to obscure temporal patterns. This hypothesis is supported by numerous studies demonstrating that the spatial variability in soil microbial communities (even across locations only a few meters apart) can be large (for example, reference 19). Another explanation is that relic DNA (legacy DNA from dead microbes that can persist in soil or secreted extracellular DNA) may dampen the observed temporal variability by effectively muting the true temporal dynamics of soil microbial communities.

While we know relic DNA is abundant in soil and other environments (20, 21), there is debate as to whether the presence of relic DNA fundamentally alters estimates of biodiversity (20, 21). Current methods do not allow us to decipher the proportion of relic DNA originating from dead cells versus the amount of extracellular DNA that may be associated with biofilms. Recent work has shown that, although relic DNA is rapidly degraded, a small portion is stabilized for extended periods of time and that environmental factors including soil temperature, moisture, and organic carbon content affect the stability of the relic DNA pool (22). Related to this, we do not have a good understanding of the age and turnover rates of relic DNA in the environment. Yet, models suggest that microbial community turnover or microscale heterogeneity in structured habitats may lead to uneven species abundance distributions in the relic DNA pool, resulting in biased estimates of microbial biodiversity (21).

**FIG 1:**
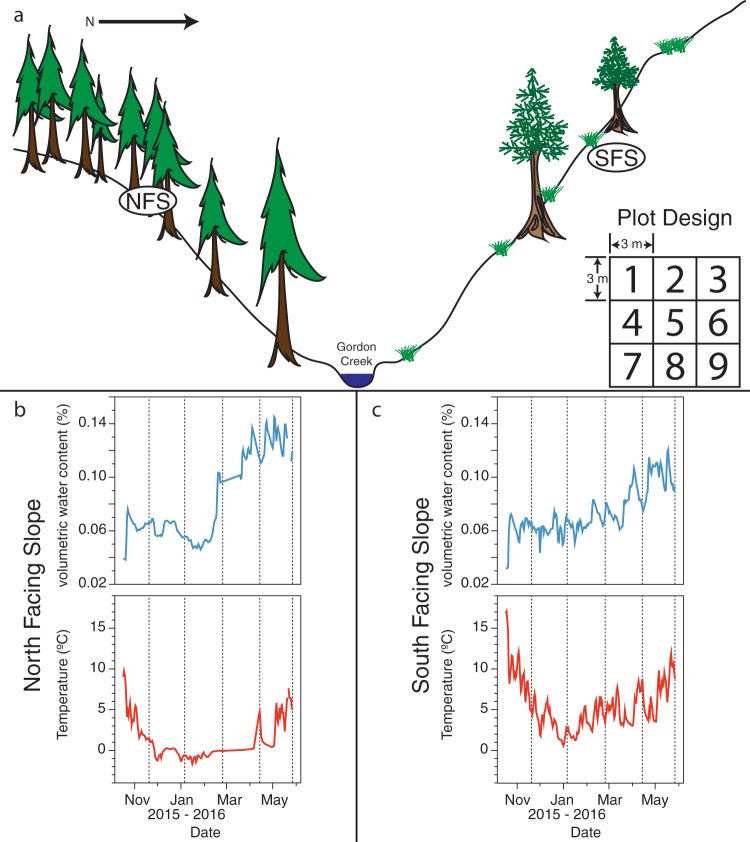
Overview of the Gordon Gulch sampling sites and environmental conditions across the sampling sites. (a) Conceptual diagram of sampling site location and plot design, adapted from reference 31. The north-facing slope (NFS) plot was centered at 40°0′44.759″N 105°28′9.123″W. The south-facing slope (SFS) plot was centered at 40°0′48.551″N 105°28′8.355″W. The inset in panel a is an illustration of the plot design. A single plot is comprised of nine 3 m × 3 m replicate subplots, as described in the main text. (b and c) Daily mean soil volumetric water content and soil temperature from *in situ* sensors at 5-cm depth for the NFS (b) and SFS (c) during the course of the experiment. Vertical dashed lines in panels b and c indicate sampling dates.

Our overarching objective was to identify groups of microbes that share temporal trajectories and to determine which environmental factors control their distributions over time. We hypothesized that ecological clusters of prokaryotes and fungi would be predictable from environmental conditions, similar to the patterns observed in aquatic systems (8, 23). To address this objective, we first needed to quantify the relative effects of spatial heterogeneity and relic DNA on temporal patterns in soil microbial communities. We chose study sites on opposing hillslope aspects of a montane ecosystem in the Boulder Creek Critical Zone Observatory (BcCZO) located within the Colorado Front Range of the Rocky Mountains. We intensively sampled two 9 m × 9 m plots, divided into 3 m × 3 m subplots every 43 to 50 days from November 2015 to May 2016 (Fig. 1; five time points total). We chose these locations because the soil microbial communities on the two hillslopes are compositionally distinct (20), relic DNA is abundant (30 to 97% of the total amplifiable prokaryotic soil DNA pool and 31 to 96% of the amplifiable fungal DNA pool [20]), and the two sites undergo distinct seasonal changes in moisture and temperature regimes (24), providing us with naturally contrasting systems in which to investigate temporal dynamics in belowground microbial communities. We characterized the microbial communities at each site using 16S rRNA gene and internal transcribed spacer 1 (ITS1) marker sequencing to profile the prokaryotic (bacterial and archaeal) and fungal communities, respectively. Here, we show the relative importance of spatial and temporal variability on soil microbial community composition and show the influence of relic DNA on these sources of variability. We use this information on temporal dynamics to identify groups of microbes that respond similarly to changes in environmental conditions—information that provides insight into the ecologies of understudied soil microbial taxa.

## RESULTS AND DISCUSSION

### Spatial variation in soil microbial communities is stronger than temporal variation and is unaffected by relic DNA.

**FIG 2:**
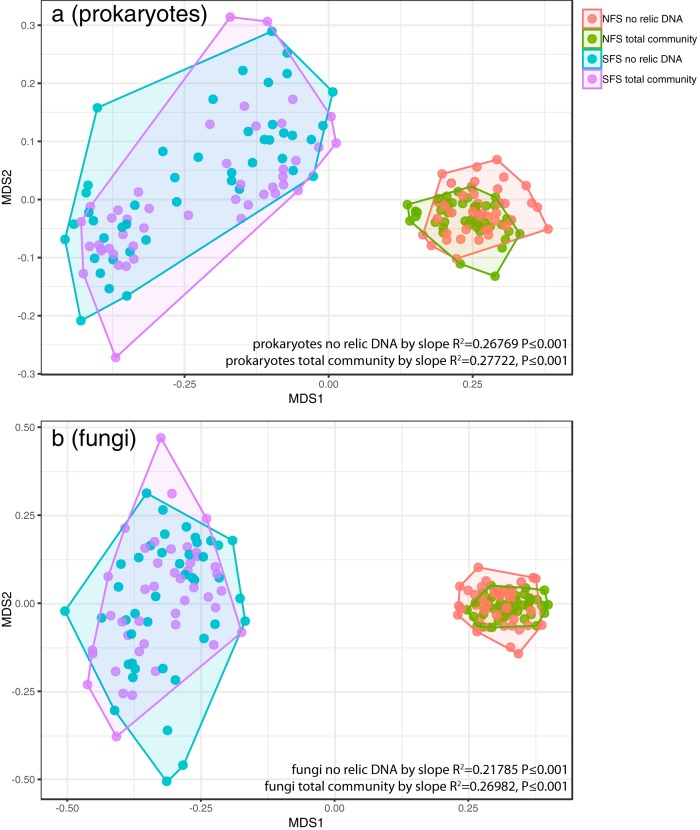
The microbial communities on the NFS are distinct from those on the SFS, regardless of time point sampled or whether relic DNA was removed. Nonmetric multidimensional scaling (NMDS) plot showing the prokaryotic (a) or fungal (b) communities for each subplot for each time point on both slopes. Points are colored by slope and whether relic DNA was removed. Hulls connect the outermost points on each slope. Permutational multivariate analysis of variance (PERMANOVA) statistics for slope differences are shown with and without relic DNA removal.

**FIG 3:**
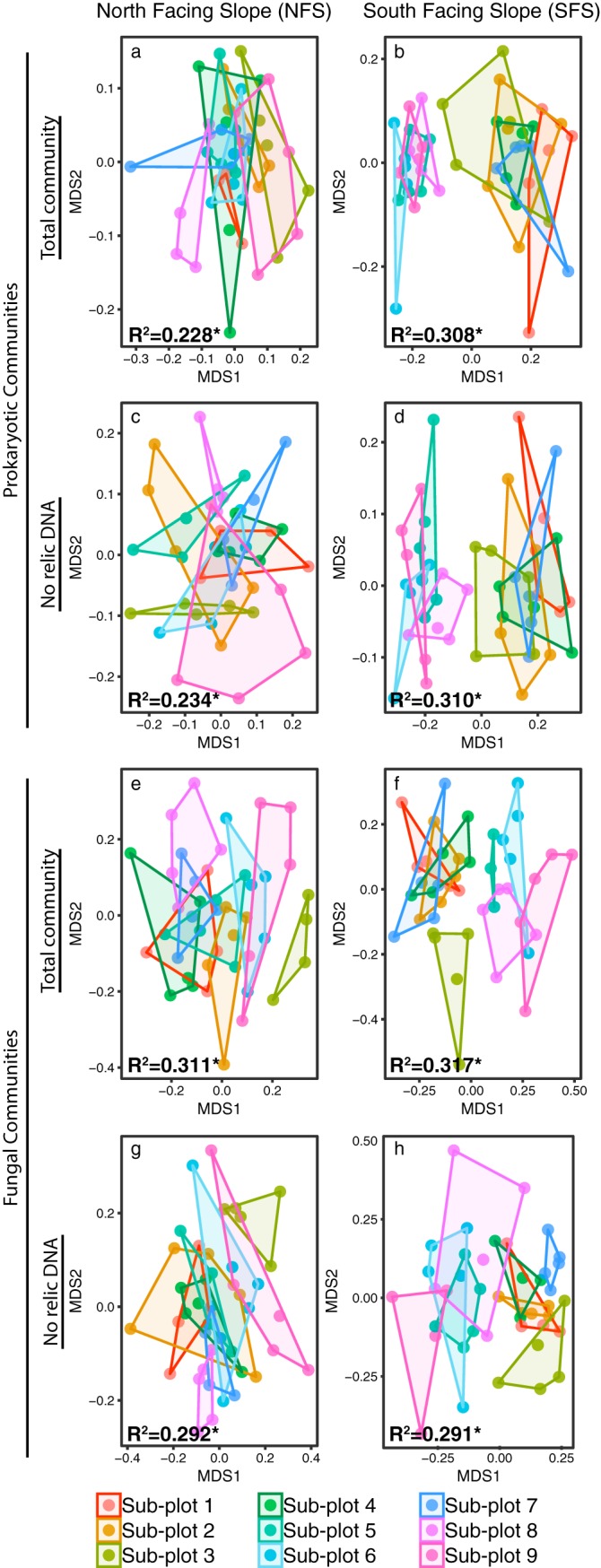
Intraplot spatial variability in soil microbial communities persists over time on both slopes regardless of whether relic DNA is removed. (a to h) NMDS plots showing the prokaryotic (a to d) or fungal (e to h) communities on the north-facing slope (a, c, e, and g) and south-facing slope (b, d, f, and h). Points represent all collected samples across the entire experiment colored by subplot number (plot layout is illustrated in Fig. 1). Hulls connect the outermost points on each slope. PERMANOVA *R*^2^ values are listed on each panel; a single asterisk indicates a PERMANOVA *P* value of ≤0.001.

Soil is a heterogeneous environment, and sampling soil microbial communities is destructive (it is impossible to collect soil twice from the same location). Thus, we first investigated the spatial variability in soil microbial community structure in the two plots. Controlling for these spatial differences is important to ensure that sufficient samples have been obtained to detect a temporal signal given that spatial variability of soil microbial communities is high, even at fine scales (19). Consistent with a previous study conducted at these sites (20), and other studies describing the spatial variability of soil microbial communities (19), the prokaryotic and fungal communities on the south-facing hillslope (SFS) were distinct from those on the north-facing hillslope (NFS), regardless of the time point sampled or whether relic DNA was removed (Fig. 2). Most notably, the SFS had higher relative abundances of the archaeal phylum *Crenarchaeota* (all of which were classified as probable ammonia-oxidizing “*Candidatus* Nitrososphaera”), and the bacterial phyla *Nitrospirae* and *Verrucomicrobia* (see Fig. S1 in the supplemental material). Beyond these expected slope-specific differences, we observed significant intraplot spatial heterogeneity in microbial community composition that persisted throughout the course of the experiment, and this intraplot heterogeneity was evident irrespective of whether relic DNA was removed. Before removing relic DNA, there was significant spatial variability across the subplots in both prokaryotic and fungal communities on the NFS (Fig. 3a and e; permutational multivariate analysis of variance [PERMANOVA] *R*^2^ = 0.228 and *R*^2^ = 0.311; *P*  ≤  0.001, respectively). These significant spatial differences were still apparent on the NFS for both prokaryotes and fungi after relic DNA was removed (Fig. 3c and g; PERMANOVA *R*^2^ = 0.234 and *R*^2^ = 0.292; *P*  ≤ 0.001, respectively). We also found significant spatial variability on the SFS in samples that were not treated to remove relic DNA, but this spatial effect was much more pronounced than on the NFS, with a clear partitioning between subplots 5, 6, 8, and 9 (see “Plot Design” in Fig. 1a for plot numbering) from the remainder of the subplots (Fig. 3b and f; PERMANOVA *R*^2^ = 0.308 and *P* ≤  0.001 for prokaryotes and *R*^2^ = 0.317 and *P*  ≤  0.001 for fungi). Similar to the NFS, these strong spatial patterns remained after relic DNA was removed (Fig. 3d and h; PERMANOVA *R*^2^ = 0.310 for prokaryotes and *R*^2^ = 0.291 for fungi; *P*  ≤ 0.001). These data show that pronounced spatial variability in soil microbial community composition at the meter scale persists over time. The presence of relic DNA does not affect our overall ability to detect this persistent spatial variation.

10.1128/mBio.02776-19.3 FIG S1 Relative abundances of prokaryotic and fungal taxa on the north-facing slope (NFS) and south-facing slope (SFS). Box plots show the distributions of the relative abundances of prokaryotic (a and b) or fungal (c and d) phyla or classes in each subplot for each time point. Box plots illustrate interquartile range ± 1.5 × interquartile range. The horizontal line in each box plot is the median. Outliers (>1.5 × interquartile range) are shown as points. Download FIG S1, PDF file, 0.4 MB. Copyright © 2020 Carini et al. 2020 Carini et al. This content is distributed under the terms of the Creative Commons Attribution 4.0 International license.

### Removing relic DNA enhanced our ability to detect temporal changes in soil microbial communities.

**FIG 4:**
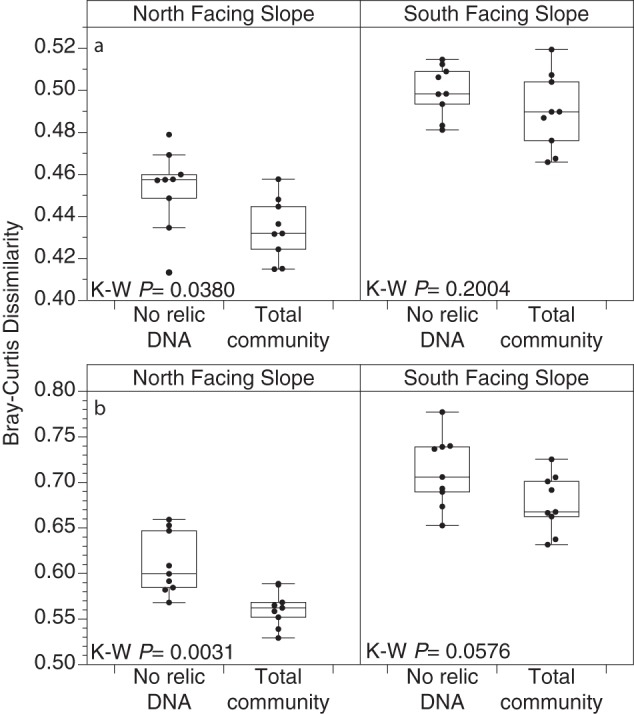
Prokaryotic microbial communities were significantly more dissimilar over time in soils without relic DNA. (a) Prokaryotes; (b) fungi. Points are the mean community dissimilarity for all pairwise Bray-Curtis distances within a subplot across all time points (*n* = 5) for samples after relic DNA removal (no relic DNA) or untreated samples (total community). Box plots illustrate interquartile range ± 1.5 × interquartile range. The horizontal line in each box plot is the median. Outliers (>1.5 × interquartile range) are shown as points outside whiskers. Kruskal-Wallis test (K-W) *P* values are shown.

We investigated the effect of relic DNA on temporal variability in belowground microbial communities on a subplot basis to control for the aforementioned high degree of intraplot spatial variability and discriminate between temporal and spatial sources of variation in microbial community structure. When limiting PERMANOVA permutations to within subplots over time, we found significant temporal variability for both prokaryotes and fungi on both slopes in untreated soils that contained the entire microbial community (Fig. S2; PERMANOVA *R*^2^ = 0.128 and *P*  ≤ 0.001 for prokaryotes and *R*^2^ = 0.124 and *P* ≤  0.001 for fungi on the NFS and *R*^2^ = 0.110 and *P*  ≤  0.001 for prokaryotes and *R*^2^ = 0.101 and *P* ≤ 0.001 for fungi on the SFS) and in soils that were treated to remove relic DNA (Fig. S2; PERMANOVA *R*^2^ = 0.119 and *P* ≤ 0.001 for prokaryotes and *R*^2^ = 0.103 and *P* ≤ 0.001 for fungi on the NFS and *R*^2^ = 0.098 and *P* ≤ 0.001 for prokaryotes and *R*^2^ = 0.106 *P* ≤ 0.001 for fungi on the SFS). We found no significant interaction between temporal variability and the presence of relic DNA, suggesting that the differences in microbial community composition between time points is not dependent on the removal of relic DNA. However, on average, the prokaryotic and fungal communities on the NFS were significantly more dissimilar over time after relic DNA was removed compared to those communities in soils that contained relic DNA (Fig. 4; Kruskal-Wallis test *P ≤ *0.05). On the SFS, the average dissimilarity over time for both prokaryotic and fungal communities was also higher after removing relic DNA, albeit the differences were not as strong as on the NFS (Fig. 4). These results show that, while compositional differences between time points can be identified in the presence of relic DNA, the removal of relic DNA can significantly enhance the ability to detect important temporal variation in the composition of soil microbial communities.

10.1128/mBio.02776-19.4 FIG S2 Significant temporal variability in soil microbial communities is present, regardless of whether relic DNA is removed. NMDS plots showing the prokaryotic (a to d) or fungal (e to h) communities on the north-facing slope (a, c, e, and g) and south-facing slope (b, d, f, and h). Points are all sample points across the entire experiment colored by time point. Hulls connect the outermost points on each slope. PERMANOVA *R*^2^ values are listed on each panel; a single asterisk indicates a PERMANOVA *P* value of ≤0.001. Download FIG S2, PDF file, 0.5 MB. Copyright © 2020 Carini et al. 2020 Carini et al. This content is distributed under the terms of the Creative Commons Attribution 4.0 International license.

### Temporal variability in distinct assemblages of prokaryotes and fungi is predictable from soil variables.

**FIG 5:**
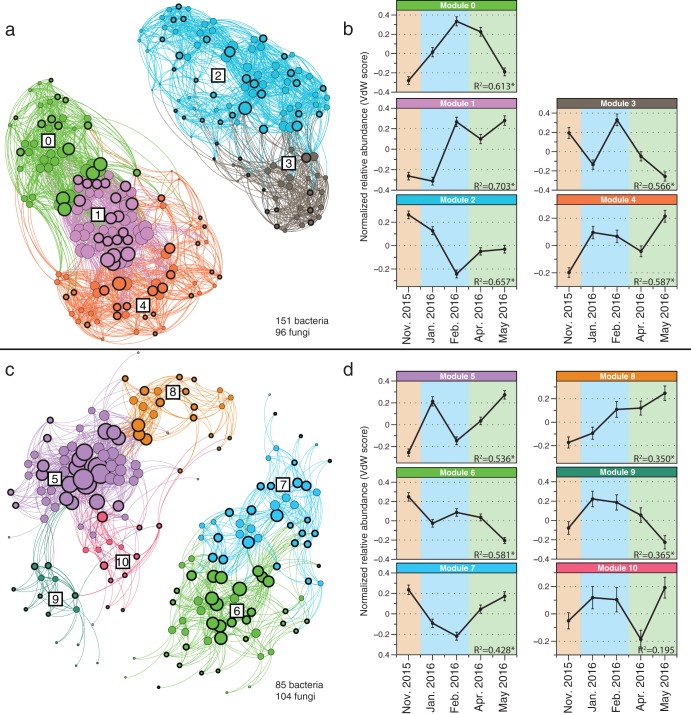
Temporal niche structure in belowground microbial communities. (a and c) Correlation networks based on significant microbe-microbe temporal correlations for the NFS (a) and SFS (c). The nodes in panels a and c are individual prokaryotic or fungal taxa. The lines between nodes represent significant (*q* value of ≤0.05) and strong (local similarity score of ≥ 0.7) positive temporal correlations across all five time points. The sizes of nodes are proportional to the number of correlations to other nodes (the degree), whereby larger nodes have more connections. Colors represent distinct modules, as determined using the modularity algorithm described in reference 26. Nodes with a thick border are fungi, and nodes with thin borders are bacteria; no archaea passed our filtering thresholds for inclusion in the figure. Boxed numbers in networks are arbitrary module numbers and match those in panels b and d. Modularity analysis of each network revealed clusters of microbes that have similar temporal patterns. (b and d) These temporal patterns were plotted for the NFS (b) and SFS (d). Points in panels b and c are the mean Van der Waerden (VdW) normalized relative abundance of all taxa in a given module. Error bars show standard errors of the means (SEM). The PERMANOVA *P* value describing the relationship of the normalized relative abundances in relation to time are shown. *P* values marked with asterisks are significant at *P* ≤ 0.005. The season is indicated by the background color as follows: orange for autumn, blue for winter, and green for spring. See Table S1 in the supplemental material for taxonomic module membership.

Characterizing shifts in the relative abundances of individual microbial taxa in temporally dynamic soil systems can give important insight into the ecologies of individual taxa and the environmental factors that influence belowground communities. Thus, we next sought to identify specific groups of taxa that exhibited correlated changes in relative abundances over time in soils after relic DNA was removed. To do this, we used local similarity analysis (LSA) (25) to identify strong (local similarity score of ≥0.7) and significant (false-discovery rate [*q* value] of ≤0.05) positive pairwise microbe-microbe temporal correlations. We constructed and analyzed networks from these correlations and extracted distinct groups (modules) of microbes from NFS and SFS networks using modularity analysis (26) (Fig. 5). On the NFS, the mean normalized relative abundances of 247 microbial taxa (151 bacteria, no archaea, and 96 fungi) were significantly correlated with at least one other taxon over time (Fig. 5a). These correlated taxa clustered into five modules, and the mean normalized relative abundances of all five modules changed significantly with time and displayed distinct temporal trajectories (Fig. 5b). On the SFS, 189 taxa (85 bacteria, no archaea, and 104 fungi) were included in the network, and clustered into six modules (Fig. 5c). The relative abundances of five of these six SFS modules changed significantly with time (Fig. 5d).

10.1128/mBio.02776-19.1 TABLE S1 Module membership by slope, including FUNGuild functional predictions for fungi. Download Table S1, XLSX file, 0.03 MB. Copyright © 2020 Carini et al. 2020 Carini et al. This content is distributed under the terms of the Creative Commons Attribution 4.0 International license.

**FIG 6:**
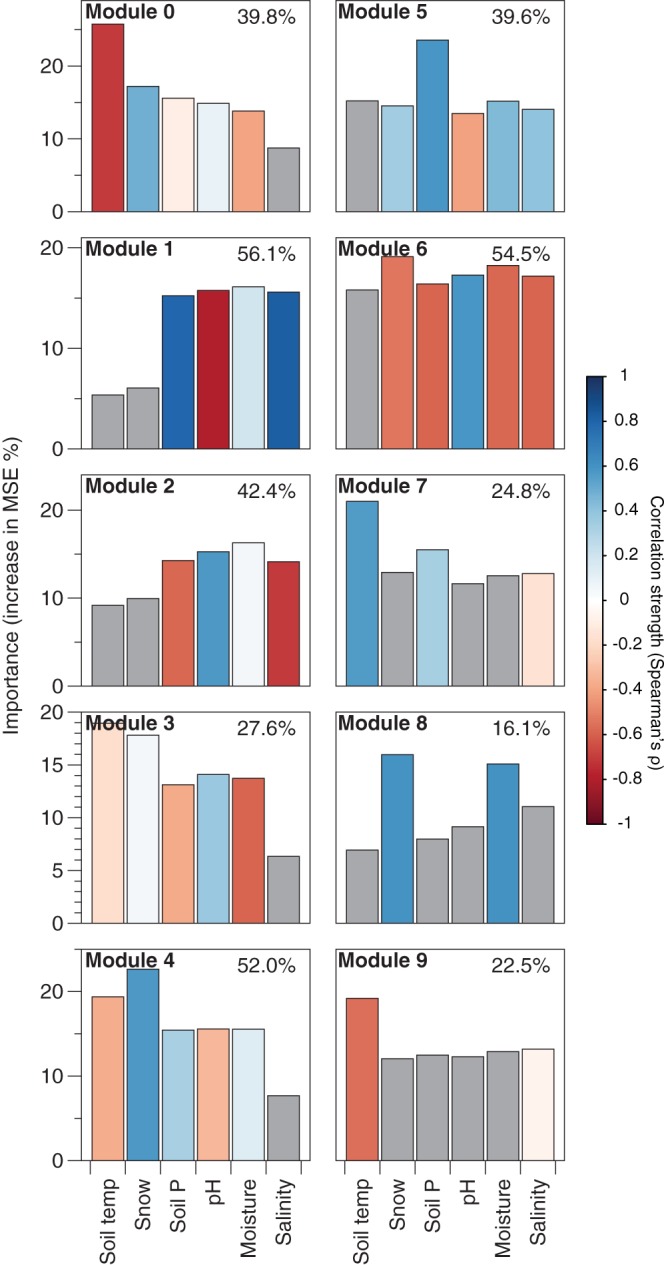
A large amount of the variation in the relative abundances of taxa within modules can be explained by temporal variation in measured soil characteristics. The percentage of temporal variation in the mean normalized relative abundances for each module that is explained by measured soil characteristics is listed in the upper right corner of each plot. Bar heights are the importance (percent increase in mean squared error [MSE]) of measured environmental and soil characteristics to the observed temporal changes in the mean normalized relative abundance of each module as determined by random forest modeling. Only environmental variables that changed significantly over time within each slope were included in the models (see Fig. S3). Module 10 was excluded from modeling because the normalized relative abundances did not change significantly with time. The color scale indicates the strength and direction of Spearman correlation between a given measured environmental variable and the mean normalized relative abundances of a given module, where +1 corresponds to a strong positive correlation and −1 corresponds to a strong negative correlation. Variables that were not significant predictors (*P* > 0.01) to the random forest models are colored gray.

A large proportion of the temporal variation in the mean normalized relative abundances of the modules that changed significantly over time could be explained by measured soil or environmental characteristics. At each time point, we measured a suite of soil and environmental parameters, including snow depth, soil temperature and moisture, extractable inorganic nitrogen (NO_3_^−^ + NH_4_^+^), salinity (electrical conductivity), extractable phosphorus (P), pH, and the chromophoric properties of water-soluble organic matter (WSOM) (a metric of organic matter lability [27]). These measured soil characteristics explained 16% to 56% of the variance in the mean normalized relative abundance of individual modules (Fig. 6). Most modules on both slopes were best predicted by climatic variables, most notably soil temperature, soil moisture, and snow depth (Fig. 6). These results are in line with previous studies demonstrating how changes in soil temperature (10, 14–16), moisture (28), and snow pack (12) can influence belowground microbial communities. In contrast, module 5 was best explained by changes in inorganic nutrient concentrations (Fig. 6). While nitrogen and phosphorus inputs can have predictable (29) and lasting (2) effects on microbial community structure, we have a more limited understanding of how short-term seasonal variation in the availability of these nutrients can influence microbial community dynamics, despite evidence that belowground microbial communities are important mediators of soil nutrient dynamics (30, 31). Our results show that a subset of soil microbes organize into modules that are responsive to these subtle changes in phosphorus availability. Variability in WSOM constituents did not contribute significantly to temporal variability in environmental conditions (Fig. S3), and thus, we excluded these measures from the models describing the temporal variability of the modules. Given that previous work at these sites showed a high degree of spatial variation in WSOM distributions (27, 32), we suspect that the pronounced spatial variability in WSOM distributions may have obscured our ability to detect significant effects of WSOM characteristics on the temporal dynamics of the soil microbial communities.

10.1128/mBio.02776-19.5 FIG S3 Results from the random forest analyses that were used to identify key environmental factors significantly correlated with time within each slope. Time was treated as a categorical variable in analyses. Out-of-bag (OOB) estimate of error shows error associated with the random forest model; 0% indicates low error. Download FIG S3, PDF file, 0.3 MB. Copyright © 2020 Carini et al. 2020 Carini et al. This content is distributed under the terms of the Creative Commons Attribution 4.0 International license.

10.1128/mBio.02776-19.6 FIG S4 Removal of relic DNA using PMA does not consistently alter the dispersion of microbial community structure. Points are pairwise Bray-Curtis dissimilarities. In each panel, all subplot and time point pairwise dissimilarities are shown for that slope (NFS [a and c] and SFS [b and d]) and group of organisms (prokaryotes [a and b] and fungi [c and d]). Box plots illustrate interquartile range ± 1.5 × interquartile range. The horizontal line in each box plot is the median. Outliers (>1.5 × interquartile range) are shown as points outside whiskers. Variance (Var) as a measure of dispersion is shown, as are Flinger-Killeen *P* values testing for homogeneity of variance between PMA-treated samples (PMA) and untreated samples (Total). Download FIG S4, PDF file, 0.9 MB. Copyright © 2020 Carini et al. 2020 Carini et al. This content is distributed under the terms of the Creative Commons Attribution 4.0 International license.

The construction of modules based on shared temporal patterns allowed us to identify biotic or abiotic factors that are correlated with shifts in the relative abundances of individual taxa. As most soil prokaryotic taxa remain undescribed (33), linking the observed temporal dynamics of specific taxa (many of which cannot be classified to the genus or species level of taxonomic classification) to their ecological attributes remains difficult. However, we did identify some bacterial taxa with temporal dynamics that can be explained from our presumed understanding of their ecologies. For example, similar to studies showing that *Bradyrhizobium* phylotypes tend to be more abundant in low pH soils (33), we found a single *Bradyrhizobium* phylotype on both slopes (modules 2 and 6) for which pH was a significant predictor (Fig. 6), indicating that temporal changes in soil pH influences the relative abundance of this abundant phylotype. Similarly, snow cover was the best predictor for the temporal variability of taxa belonging to module 4 (Fig. 6). We identified several taxa in module 4 that have been directly linked to the microbial communities associated with snow, including the bacterial phylotypes classified as *Herminiimonas* sp. and *Sphingobacteriaceae* spp. (34, 35) (see Table S1 in the supplemental material). We also observed several fungal phylotypes belonging to the Mucorales and Mortierellales orders that clustered in module 4 (Table S1). Members of these fungal groups have been termed “snow-molds” and are commonly observed on the surface of soil during snowmelt at these sites (36).

Our study also provides insight into the short-term temporal variation of ectomycorrhizal communities, the environmental factors that influence these patterns, and other fungal and prokaryotic taxa that covary with ectomycorrhizal fungi. Ectomycorrhizal fungi were found on both slopes and partitioned into several modules that were significantly variable over time (on the NFS, modules 0 to 4 and on the SFS, modules 6, 7, and 9; Fig. 5 and Table S1). On the NFS, 56% of the predicted ectomycorrhizal fungal taxa were found in module 2 (Fig. 5 and Table S1). Module 2 was best predicted by soil moisture and pH, suggesting that these ectomycorrhizal taxa prefer slightly drier and higher pH soils (Fig. 6). However, other ectomycorrhizal taxa on the NFS were best predicted by other combinations of soil characteristics and environmental parameters, suggesting a degree of niche partitioning within soil ectomycorrhizal fungal communities (Fig. 6), a finding in agreement with previous observations (37, 38). Fewer ectomycorrhizal taxa displayed correlated behavior with other microbes on the SFS, but the majority of these taxa (69%) belonged to module 6, which was best predicted by snow and moisture. On the SFS, these ectomycorrhizal taxa tended to be more abundant when soils were drier with less snow cover (Fig. 6). These findings indicate a degree of temporal niche partitioning in ectomycorrhizal fungal communities on both slopes in response to distinct environmental conditions (Fig. 6).

### Conclusions.

We show that prokaryotes and fungi living in surface soils at the two sites studied here are dynamic over time and that a more detailed characterization of the temporal variability in soil microbial communities is critical to understanding the dynamic nature of the soil microbiome. The extensive spatial and temporal sampling design of our study allowed us to disentangle the relationships between spatial heterogeneity in microbial communities, temporal dynamics of these communities, and the effect of relic DNA on these temporal patterns. Unsurprisingly, spatial variation in community structure at both the hillslope scale and the meter scale (intraplot) was the dominant source of variability at these sites and relic DNA had no significant effect on our ability to characterize these spatial patterns (Fig. 2 and 3).

When controlling for this spatial variability, we were able to detect significant temporal shifts in microbial community composition at these sites, regardless of whether relic DNA was removed or not. We emphasize that the magnitude of the temporal variation in soil microbial communities was consistently lower than the spatial variation, even between subplots located only a few meters apart. This spatial variability in surface soil microbial communities was relatively stable over time in the study plots, suggesting that efforts to describe spatial variation in overall community composition are not necessarily impacted by collecting samples across different time points.

We show that when sites are sampled sufficiently across space, temporal variability is apparent both in soils that have been treated to remove relic DNA and in untreated samples. However, we provide new evidence that the removal of relic DNA from soil results in greater dissimilarity over time, suggesting that by removing relic DNA, we enhance our ability to detect temporal patterns in the belowground communities (Fig. 4). These findings support our previous hypothesis (20), and predictions based on modeling (21), that the presence of relic DNA can hinder the ability to detect temporal patterns in dynamic belowground soil microbial communities. The presence of relic DNA, even in large amounts, does not automatically lead to relic DNA biases in other ecosystems (21). However, our data do suggest that relic DNA has important effects on studies of temporal variation in soil microbial communities (and possibly in other ecosystems) and that the consequences of failing to remove relic DNA would not be apparent from single time point samples. These findings are consistent with other work suggesting that relic DNA may affect microbial community dynamics over time (22). Thus, it remains challenging to know *a priori* how relic DNA might influence measurements of microbial community structure for a given study, as there are several unknown variables that contribute to relic DNA dynamics in soil. First, the bulk growth rates of microbes *in situ* and how growth rates vary across taxa (both fungal and prokaryotic) are poorly understood and likely soil and condition specific. Second, although previous studies have suggested that relic DNA likely degrades at a constant rate (21), we do not have information on the rates of *in situ* relic DNA generation—rates that may be proportional to microbial death or community turnover rates. These two factors are necessary to calculate the residence time of the relic DNA pool in soil. Our study is the first to demonstrate that relic DNA may affect our interpretation of the strength of temporal dynamics in soil microbial communities, highlighting a need for future studies that focus on understanding the residence time of relic DNA under different conditions.

The belowground environment is one of the most complex and dynamic microbial habitats on Earth. By controlling for spatial and relic DNA effects on temporal variability in these soil microbial communities, we identified groups of microbes that have similar temporal dynamics and the environmental factors that predicted their temporal distributions. A deeper understanding of relationships between soil microbiota can help resolve both the roles of individual taxa and potential “ecological clusters” with emergent function. For example, taxa that covary may exhibit similar niche preferences and compete for growth substrates. In contrast, taxa belonging to a given module may broadly respond to similar environmental signals but occupy distinct substrate niches (39). Microbes that are correlated over time may interact through cross-feeding of metabolic substrates or coutilization of leaky functions (40)—either directly or in a time-lagged manner. Understanding the basis for shared temporal dynamics is important, as microbial interactions are crucial in shaping microbial communities (41) but difficult to measure directly (42). Future investigations that combine cell culturing, synthetic microbial communities, and genomics may help resolve the specific drivers of these cooccurrence patterns (39, 43, 44).

## MATERIALS AND METHODS

### Site description, plot design, and sampling procedure.

The two plots were set up on opposing slopes alongside an instrumented transect at ∼2,530-m elevation (approximately 40.01°N, 105.47°W), chosen on the expectation that there would be a high level of temporal variability in soil microbial communities as a result of intra-annual changes in soil moisture and temperature (24). The north-facing slope (NFS) and south-facing slope (SFS) have distinct soil and vegetation characteristics and experience different water delivery patterns, particularly during snowmelt (24) (Fig. 1). The NFS and SFS soils are Ustic dystrocryept (Catamount series) and Lithic haplstoll, respectively (45). Soil moisture and temperature were variable over the course of the study and followed expected seasonal trends (Fig. 1). In general, the NFS had a higher soil moisture and a lower temperature than the SFS (Fig. 1). The NFS is vegetated with moderately dense *Pinus contorta* (Lodgepole pines) and develops a snowpack during the winter that melts in spring. In contrast, the SFS is much more sparsely vegetated with *Pinus ponderosa* (Ponderosa pines), intervening grasses, and *Arctostaphylos uva-ursi* (kinnikinnick) shrubs and experiences pulses of snowmelt throughout the winter and spring. We sampled 10 to 15 soil cores (0 to 5 cm, mineral soils only; 1-inch core diameter) at randomly selected locations within each subplot at each of the five time points. The soil cores from each subplot were pooled, sieved to 2 mm, and homogenized at each time point and partitioned for microbial community and nutrient analyses. Sample dates are reported in Table S2 in the supplemental material.

10.1128/mBio.02776-19.2 TABLE S2 Time points, sampling dates, days between time points, and season. Download Table S2, CSV file, 0.00 MB. Copyright © 2020 Carini et al. 2020 Carini et al. This content is distributed under the terms of the Creative Commons Attribution 4.0 International license.

### Continuous environmental measurements.

Several automated measurements were collected every 10 min at a meteorological station located near the sample sites (see “Data availability” below for data source information). Each slope was instrumented with a soil temperature sensor (Campbell Scientific T-107 temperature probe), and a soil water content reflectometer (Campbell Scientific CS616) located 5 cm below ground. The daily averages from these sensors on each slope are illustrated in Fig. 1b and c. When modeling the relative mean importance of temperature and volumetric water content to module temporal distributions, we used the average of daily mean values from these sensors between sample dates, except for the first time point, which is the mean from the preceding 34 days. Snow depth was measured using digital ultrasonic snow depth sensors (Judd Communications Inc.) fitted with CR1000 dataloggers (Campbell Scientific). Snow depth is reported as mean daily snow depth between sampling points from three sensors on each slope (NFS at snow pole 3, sensors 1 to 3 and SFS snow pole 10, sensors 9, 11, and 15).

### Discrete environmental measurements.

Inorganic N pools were measured for each subplot at each time point except for the January 2016 sample on the NFS, subplots 1 and 2 and SFS subplot 3, where insufficient soil was collected. Sieved soils for inorganic N analyses were stored at 4°C for <72 h. Inorganic N pools were extracted from 10 g field soil (moist) in 100 ml of 2 M potassium chloride with shaking by hand for 20 s every 3 h for 18 h and then filtered through cellulose Whatman 1 filters. Ammonium (NH_4_^+^) concentrations were measured in these extracts on a BioTek Synergy 2 microplate reader with a detection limit of 0.009 mg N liter^−1^, and nitrate (NO_3_^−^) concentrations were measured on an OI Analytical FS-IV analyzer with a detection limit of 0.5603 μg N liter^-1^. Dissolved inorganic nitrogen (DIN) was calculated as the sum of NH_4_^+^ and NO_3_^−^.

Water-soluble organic matter (WSOM) was analyzed for each subplot at each time point except for the following plots, where insufficient sample was collected: NFS February 2016 (all subplots); SFS February 2016 subplots 1, 8, and 9 and April 2016 subplot 5. Sieved soils were stored at –20°C until WSOM extraction. WSOM was extracted by leaching 10 g of soil with 50 ml of 0.5 M K_2_SO_4_ following the methods described in reference 27. The spectroscopically active portion of the WSOM was characterized with UV-visible (UV-Vis) and fluorescence spectroscopy. Samples were diluted to minimize the inner filter effect (46), and the UV-Vis absorbance was measured from 200 to 800 nm in 1-nm increments using an Agilent 8453 spectrophotometer with a 1-cm path length. Dissolved organic carbon (DOC) and total nitrogen were measured on a Shimadzu TOC-V total organic carbon analyzer. SUVA_254_, a proxy for the aromaticity of the WSOM, was calculated as the absorbance at 254 nm normalized by the DOC concentration (47). Fluorescence scans were collected on a Horiba Jobin Yvon Fluoromax-4 spectrofluorometer with a 1-cm quartz cuvette and normalized to Raman units (48). The fluorescence index (FI) (49) and humification index (HIX) (50) were calculated from the fluorescence scans using parallel factor analysis (PARAFAC) to further resolve discrete components representing different classes of fluorophores (27).

Other standard soil characteristics were measured at each time point by pooling equal masses of soil from each subplot plot on each slope. These measurements included pH, electrical conductivity (milliMhos per centimeter), and P (parts per million). Standard soil chemical analyses were performed at the Colorado State University Soil Water and Plant Testing Laboratory using their standard protocols.

### Relic DNA removal and DNA extraction.

Relic DNA was removed using a propidium monoazide (PMA) treatment as described previously (20). PMA is a DNA-intercalating photoreactive chemical that binds extracellular DNA and DNA in cells with compromised cell membranes. PMA irreversibly binds to and destroys DNA after exposure to bright light. We previously reported on the efficacy and limitations of this approach in a wide range of soil types (20). Briefly, 0.03 g of each pooled soil sample from each subplot was subsampled, resuspended in 3.0 ml phosphate-buffered saline (PBS) (1% [wt/vol] slurry) and either treated with 40 μM PMA in the dark or left untreated. Both treated and untreated samples were vortexed in the dark for 4 min and exposed to a 650-W light for four 30-s light/30-s dark cycles to activate PMA in treated samples. Light-exposed samples were frozen at –20°C until DNA extraction. DNA was extracted from 800 μl of PMA-treated and untreated soil slurries using a PowerSoil-htp 96-well soil DNA isolation kit (MoBio) following the manufacturer’s instructions, except that 770 μl was used in the C2 step. All samples and “no soil” negative controls were randomized into these 96-well DNA extraction plates and extracted simultaneously.

### Amplicon sequencing and analytical methods.

For sequence-based analyses of 16S rRNA and internal transcribed spacer (ITS) marker regions, we used the approaches described previously (20). Briefly, we amplified each sample in duplicate in 25-μl PCR mixtures containing the following: 12.5 μl of Promega GoTaq Hot Start Colorless Master Mix; 0.5 μl of each barcoded primer (10 μM [each] bacterial 16S, 515F [5′-GTGCCAGCMGCCGCGGTAA-3′] and 806R [5′-GGACTACHVGGGTWTCTAAT-3′]; fungal ITS [5′-CTTGGTCATTTAGAGGAAGTAA-3′] and ITS2 [5′-GCTGCGTTCTTCATCGATGC-3′]); 10.5 μl water; 1 μl of template DNA. The thermal cycler program was as follows: (i) 94°C for 5 min; (ii) 35 cycles, with 1 cycle consisting of 94°C for 45 s, 60 s at 50°C, and 72°C for 90 s; and (iii) a final extension step of 72°C at 10 min. Duplicate PCR mixtures for each sample were pooled, cleaned, and normalized using the ThermoFisher Scientific SequalPrep normalization plate kit. Cleaned and normalized amplicons were pooled, spiked with 15% phiX, and sequenced on an Illumina MiSeq using v2 500-cycle paired-end kits. The samples were sequenced in two batches total—one for prokaryotes and one for fungi. Reads were processed as described in reference 29. Briefly, raw amplicon sequences were demultiplexed according to the raw barcodes and processed with the UPARSE pipeline (51). A database of ≥97% similar sequence clusters was constructed in USEARCH (version 8) (52) by merging paired-end reads, using a “maxee” value of 0.5 when quality filtering sequences, dereplicating identical sequences, removing singleton sequences, clustering sequences after singleton removal, and filtering out cluster representative sequences that were not ≥75% similar to any sequence in Greengenes (for prokaryotes; version 13_8) (53) or UNITE (for fungi) (54) databases. Demultiplexed sequences were mapped against the *de novo*-constructed databases to generate counts of sequences matching clusters (i.e., taxa) for each sample. Taxonomy was assigned to each taxon using the RDP classifier with a threshold of 0.5 (55) and trained on the Greengenes or UNITE databases. To normalize the sequencing depth across samples, samples were rarefied to 10,159 and 7,076 sequences per sample for the 16S rRNA and ITS analyses, respectively. “No soil” negative controls on the prokaryotic sequencing run contained between 86 and 870 reads (*n* = 10 median = 314 reads). “No soil” negative controls on the fungal sequencing run contained between 1 and 503 reads (*n* = 10 median = 6.5 reads). Fungal taxa were assigned to broad ecological categories using FUNGuild (56). Bray-Curtis distances were calculated on square root-transformed taxon relative abundances using the mctoolsr R package (57).

### Effect of relic DNA removal on the dispersion of microbial community structure.

We compared the dispersion in pairwise Bray-Curtis distances among untreated soils (which contain relic DNA) versus those soils treated to remove relic DNA. We conducted this analysis across the entire data set (Fig. S4) and by subplot or time point (Fig. S5). The magnitude and direction of dispersion effects after PMA treatment were variable, indicating that the removal of relic DNA does not systematically affect the dispersion in microbial community structure. Thus, there is no consistent effect of using PMA to remove relic DNA on the variance in microbial community structure.

10.1128/mBio.02776-19.7 FIG S5 Comparison of pairwise distances by treatment (PMA treated or untreated) and time point or subplot. Points are pairwise Bray-Curtis dissimilarities. Panels a to d show prokaryotic dissimilarities for the NFS (a and c) and SFS (b and d) separated by treatment (treated with PMA to remove relic DNA [PMA] or untreated total community samples [Tot]) and either time point (a and b) or subplot (c and d). Panels e to h show fungal dissimilarities for the NFS (e and g) and SFS (f and h) separated by treatment and either time point (e and f) or subplot (g and h). Box plots illustrate interquartile range ± 1.5 × interquartile range. The horizontal line in each box plot is the median. Outliers (>1.5 × interquartile range) are shown as points outside whiskers. Flinger-Killeen *P* values testing for homogeneity of variance (as a measure of dispersion) between PMA-treated samples and untreated samples as a function of time point (a, b, e, and f) or subplot (c, d, g, and h) are shown. Download FIG S5, PDF file, 0.7 MB. Copyright © 2020 Carini et al. 2020 Carini et al. This content is distributed under the terms of the Creative Commons Attribution 4.0 International license.

### Temporal analyses and network construction.

We identified significant temporal correlations in the relative abundances of individual taxa on each slope that were, on average, ≥0.1% of the community across all samples in soils that were treated to remove relic DNA using extended local similarity analysis (eLSA) (25) with the following parameters: lsa_compute -s 5 -r 9 -p perm. We defined significant temporal associations as those with a local similarity (LS) score of ≥ 0.7 (i.e., strong to very strong correlations) and a false-discovery rate (*q* value) of ≤0.05. Pairs of significantly correlated taxa were analyzed in Gephi (version 0.8.2). Network modularity was calculated by implementing the “modularity” function (26) within Gephi, with a resolution setting of 0.9 for both slopes. Node identifiers (IDs) (individual taxa) belonging to the same module were extracted to delineate temporal patterns in their normalized relative abundances. Van Der Waerden (VdW) normalization (normal scores) converts data ranks to quantiles of a standard normal distribution. In eLSA, data are VdW normalized prior to analysis. We obtained VdW scores for each node ID for Fig. 5b and d using the tRank command in the multic R package (58).

### Random forest analysis.

For each slope, we used two rounds of random forest modeling (59) to first identify the measured environmental and soil variables that were significant predictors of time (*P* ≤ 0.05), using time as a response variable (Fig. S3). Only these significant environmental factors were used to predict changes in module abundance over time. We conducted a second round of random forests analysis with the significant environmental predictors shown in Fig. S3 to identify the most important environmental factors or soil characteristics that predicted the mean normalized relative abundances of each module (see reference 60 for a similar approach). The importance (increase in mean square error percentage) and significance of each predictor was computed for each tree and averaged over the forest (9,999 trees) using the rfPermute R package. Significant predictors were defined as those with a *P* value of ≤0.05. Samples for which environmental and soil characteristics were missing because of insufficient sample were excluded from the random forest models. We used Spearman correlation analysis in Fig. 6 to describe the direction and strength of the relationships (positive or negative) between mean normalized module abundances and the environmental variables. Spearman correlations were conducted in R with the hmisc package (61).

### Data availability.

Raw DNA sequence data, the corresponding map file, soil and environmental characteristics, and R code used to process the data are available on figshare.com (https://figshare.com/articles/Code_used_in_analyses/9961619 and https://figshare.com/articles/Cross-domain_temporal_seq_files_zip/6710087). Snow depth data are available through the Boulder Creek Critical Zone Observatory website http://criticalzone.org/boulder/data/dataset/2423/. Temperature data for the NFS and SFS are available through the Boulder Creek Critical Zone Observatory website http://criticalzone.org/boulder/data/dataset/2426/.
