# Supplementary figures and images for: Effects of Spatial Variability and Relic DNA Removal on the Detection of Temporal Dynamics in Soil Microbial Communities
Source: mBio. 2020 Jan 21;11(1):e02776-19. doi: 10.1128/mBio.02776-19 (PMC6974563; doi:10.1128/mBio.02776-19)

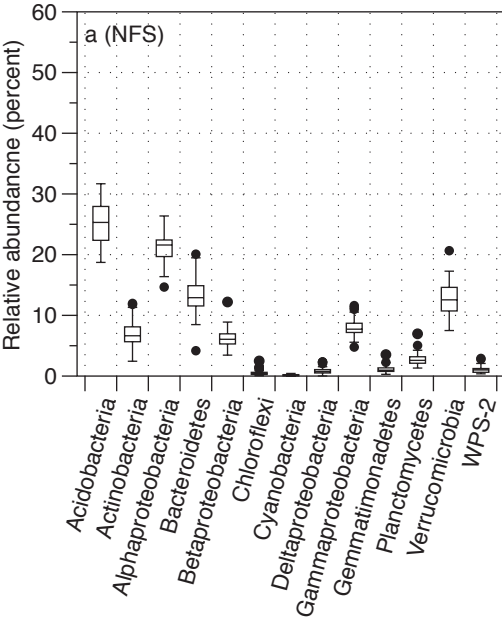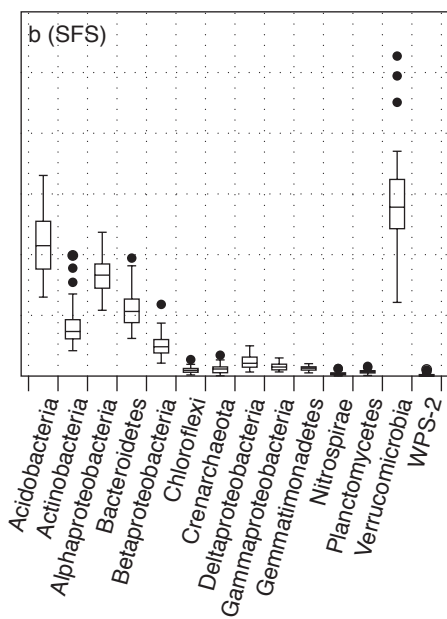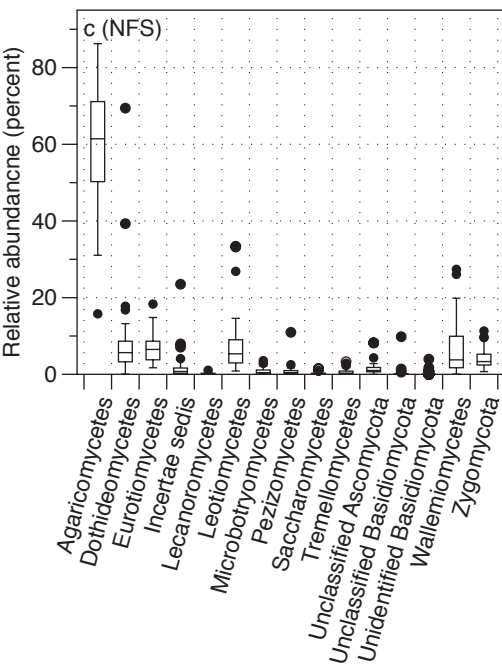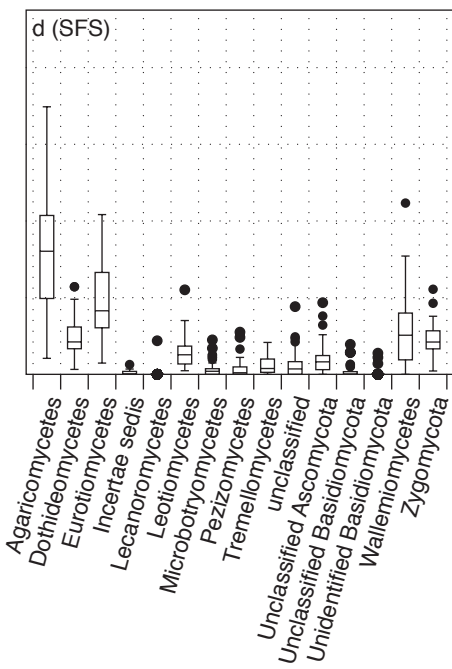

Supplement: FIG S1 [file mBio.02776-19-sf001.pdf]

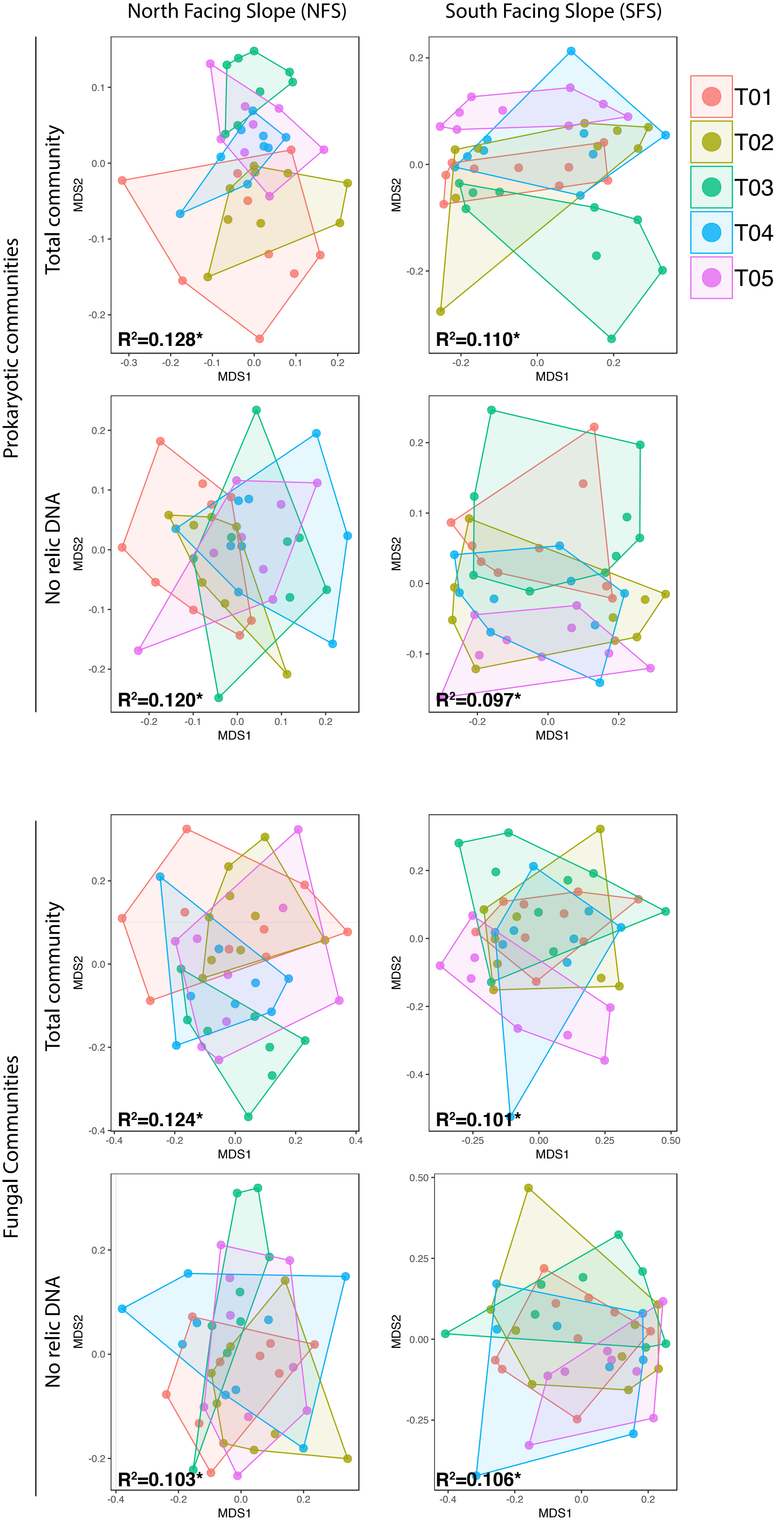

Supplement: FIG S2 [file mBio.02776-19-sf002.pdf]

North Facing Slope (NFS)

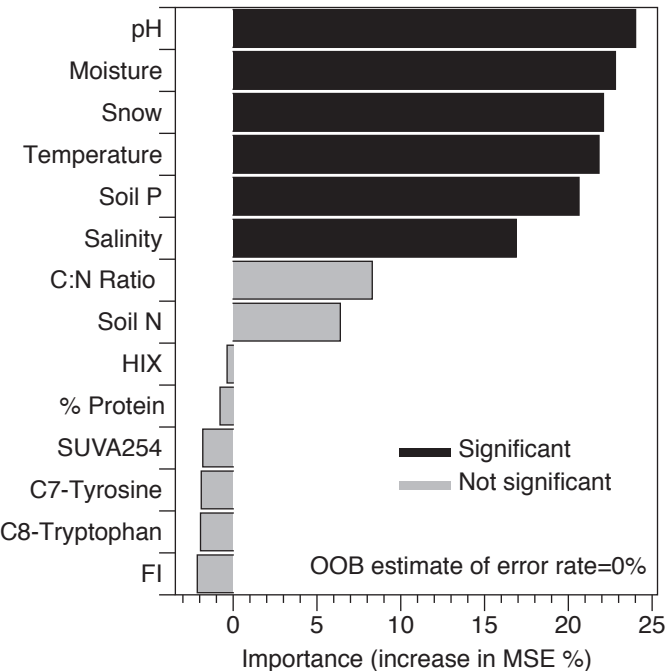

South Facing Slope (SFS)

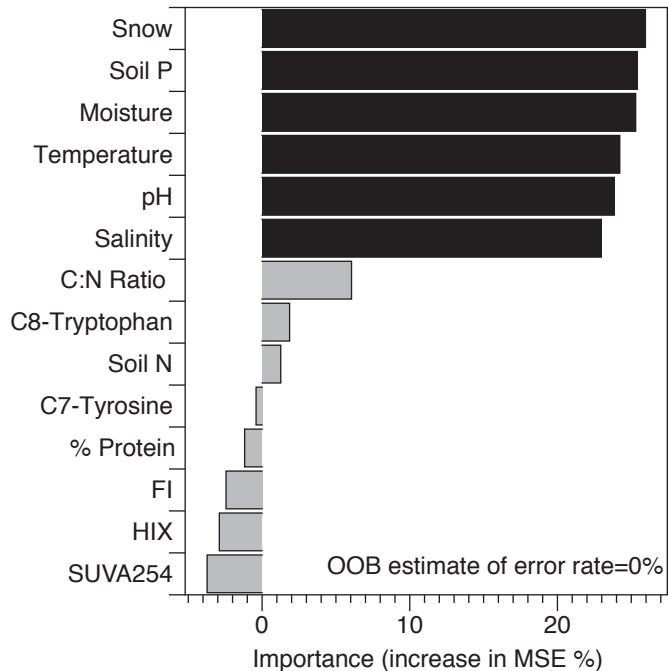

Supplement: FIG S3 [file mBio.02776-19-sf003.pdf]

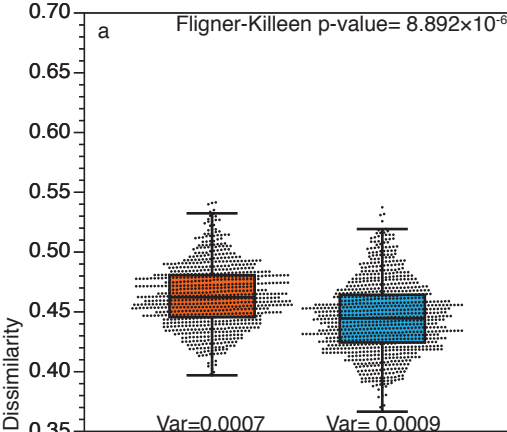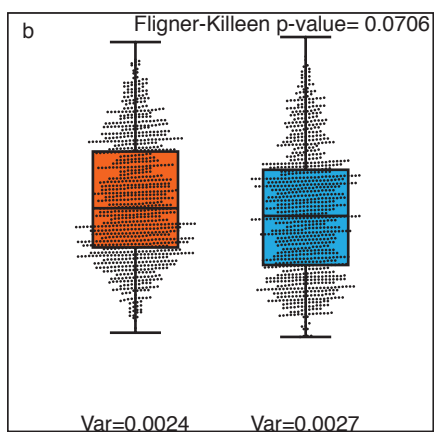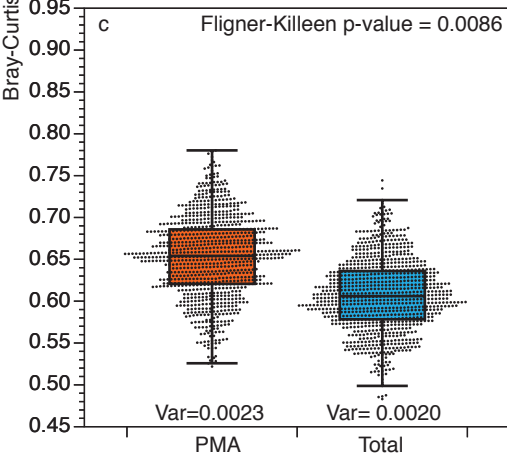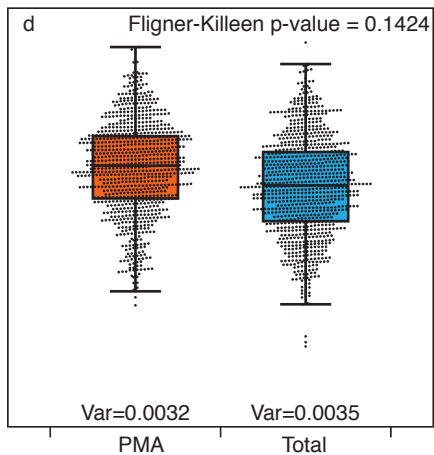

Supplement: FIG S4 [file mBio.02776-19-sf004.pdf]

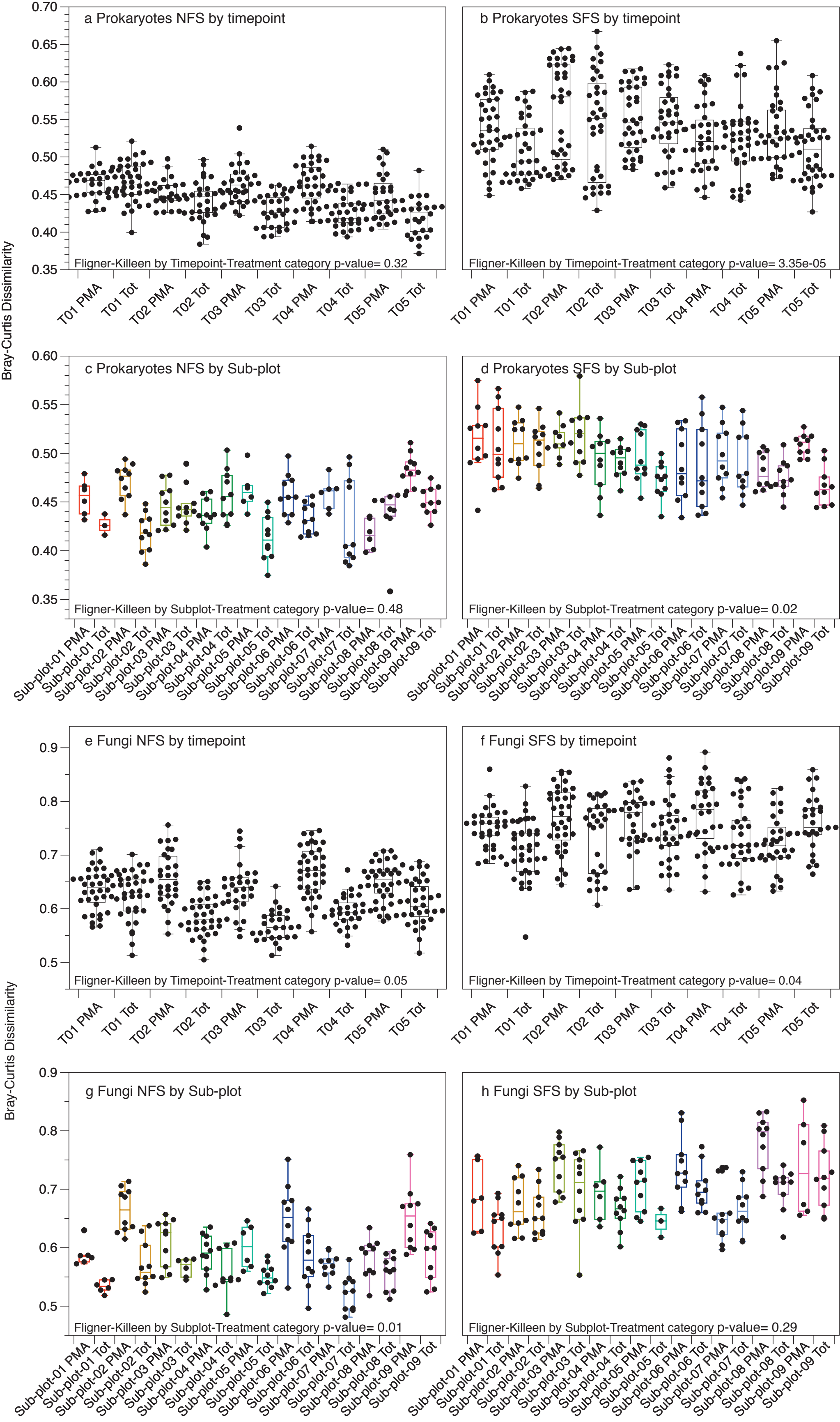

Supplement: FIG S5 [file mBio.02776-19-sf005.pdf]
